# Case Report: Death penalty versus indefinite imprisonment in Japan: a case note of two court judgments involving autism spectrum disorder and autistic traits

**DOI:** 10.3389/fpsyt.2025.1690300

**Published:** 2026-01-19

**Authors:** Hiroko Kashiwagi, Naotsugu Hirabayashi

**Affiliations:** Department of Forensic Psychiatry, National Center Hospital of Neurology and Psychiatry, Tokyo, Japan

**Keywords:** autism spectrum disorder, death penalty, judicial decision-making, rehabilitation, remorse

## Abstract

The World Psychiatric Association condemns the sentencing to death and execution of individuals with mental illness or intellectual and developmental disabilities. However, in Japan, death sentences have been confirmed in individuals diagnosed with delusional disorders and autism spectrum disorder (ASD). We report two Japanese court cases in which medical professionals with ASD or autistic traits committed multiple homicides. Although ASD characteristics were acknowledged, one defendant received the death penalty while the other was sentenced to indefinite imprisonment. This case note aims to analyze how psychiatric evaluations and judicial reasoning distinguished between these two outcomes. We highlight the reliance on subjective assessments of remorse and the limited standardization in evaluating rehabilitation potential when distinguishing between the death penalty and indefinite imprisonment in defendants with ASD traits. Psychiatric expertise should contribute to fairer and more evidence-based sentencing.

## 1 Introduction

At the 23rd World Congress of Psychiatry held in September 2023, the World Psychiatric Association declared that individuals with mental illness or intellectual and developmental disabilities should not be sentenced to death or executed (1). However, in recent years, death sentences have been confirmed in individuals diagnosed with delusional disorders or autism spectrum disorder (ASD) during psychiatric evaluations in Japan (2, 3).

Currently, there is no evidence suggesting that individuals with ASD are more likely to commit violent crimes than the general population; however, in rare cases, they may commit crimes in extreme forms that attract public attention. In Japan, in 2014 and 2016, mass murder incidents were committed by medical professionals with autistic traits at their workplaces, and the death penalty was sought for both perpetrators.

## 2 Objective

We report two Japanese court cases in which medical professionals with ASD or autistic traits committed multiple homicides. Although ASD characteristics were acknowledged, one defendant received the death penalty while the other was sentenced to indefinite imprisonment. This case note aims to analyze how psychiatric evaluations and judicial reasoning distinguished between these two outcomes.

## 3 Methods

The descriptions here do not go beyond the content of the trial proceedings and judgments. Both cases were tested by a lay judge panel in the first instance, and the first-instance judgment was upheld in the appeal trial. In both cases, the court’s decision is final. The analysis was based on the contents of the published judgment. The cases were primarily collected based on court precedents provided through the online services of TKC Law Library. TKC Law Library compiles precedents into a database and provides access to judgments for academic use on a paid basis. While judgment texts are not subject to copyright under Article 13 of the Copyright Act, written permission was obtained from the company to quote the judgment texts. Additionally, when citing judgment texts, we have included the reference numbers from the LEX/DB database.

The strength of this case report lies in the fact that it is based on facts established in court. Its limitation is that the basis for the symptoms and diagnosis, as well as the basis for the connection between the symptoms and the crime, are solely based on the court’s findings in response to the forensic psychiatric report presented in court. Furthermore, the final judgment was influenced by various other legal and customary factors not mentioned here.

In this study, each judgment was systematically reviewed according to a predefined sequence of analytical categories: (i) symptoms described in the judgment, (ii) diagnostic conclusions, (iii) the court’s description of how both the defendant’s psychiatric disorders and their intact or non-disordered psychological characteristics influenced the mechanism of the offense, (iv) the evaluation of criminal responsibility, and (v) the reasoning underlying the sentencing decision.

In Japan, criminal responsibility is evaluated according to two components: (i) the capacity to appreciate the rightfulness or wrongfulness of one’s actions (cognitive capacity), and (ii) the capacity to act in accordance with such appreciation (volitional capacity). Full responsibility is recognized when both capacities are preserved. Diminished responsibility is found when either capacity is markedly impaired. Legal insanity is determined when either capacity is lost.

### 3.1 Case description

#### 3.1.1 Case 1

A man in his 20s working at a nursing home as a caregiver performed cardiopulmonary resuscitation (CPR) after three residents of the nursing home fell to their deaths from a balcony (4, 5). Over two months, he killed three residents by throwing them off a fourth- or sixth-floor balcony. He was under considerable mental stress, had to deal with sudden incidents that were not part of his job, verbal abuse, and violence, and was reminded of a time in the past when his father had pointed a kitchen knife at him. He threw the first resident off the balcony and killed him. He subsequently performed CPR on the victim. He had been passionate about first aid since childhood. Obsessed with performing chest compressions and wanting to be seen by others, he killed his second and third victims by throwing them off the balcony and having them hit the ground, subsequently performing chest compressions on them. During the appeal trial, the motive of wanting to be praised for performing CPR was denied.

He was diagnosed with ASD in accordance with the Diagnostic and Statistical Manual of Mental Disorders, 5^th^ edition (DSM-5) criteria. His IQ was low at 68, but his qualifications as an emergency medical technician and academic achievements ruled out a diagnosis of intellectual disability. In the first trial, it was determined that the characteristics of ASD, such as the pain he felt from sudden responses, the pain he felt from being subjected to verbal abuse and violence, and his obsession with CPR, had an impact on his actions.

The judgment also identified multiple non-psychiatric factors that contributed to its conclusions regarding criminal responsibility and sentencing. Although the influence of ASD was acknowledged, the court characterized the degree of impairment as relatively mild. At the same time, the judgment noted numerous aspects of the defendant’s conduct that reflected rational and purposeful behavior consistent with a pre-existing intent to kill, including checking the movements of other night-shift staff and taking steps to conceal evidence.

The court observed that the defendant clearly understood that his actions constituted criminal behavior and acted with full awareness of their wrongfulness. It held that there was no basis to conclude that he lacked the ability to distinguish right from wrong, or that it was markedly difficult for him to refrain from committing the offenses, and therefore found that he possessed full criminal responsibility.

Furthermore, the judgment described the killings as extremely cruel, noting that the defendant threw the residents “as if throwing objects,” demonstrating a strong intent to kill and an absence of humanity. The court also emphasized that the defendant created false records and attempted to make the deaths appear as suicides, thereby worsening the circumstances of the crimes, and that he betrayed the trust inherent in his role as a caregiver.

Regarding the sentence, it was said that the cold-heartedness of the crime (which showed no sign of humanity), the fact that it appeared to have been premeditated, and the fact that he denied each crime and was preoccupied with irrational excuses, showed that he had no remorse. Therefore, he did not reach the starting point of rehabilitation, and there were few circumstances that could have led to rehabilitation. He was sentenced to death as requested by the prosecutor.

#### 3.1.2 Case 2

A woman in her 30s, who worked as a nurse, killed three people by mixing benzalkonium chloride in their intravenous drips at a hospital, and then added benzalkonium chloride in the drips of four other hospitalized patients (6, 7). At a hospital where she used to work, she had a difficult time when she was blamed by families for not being able to handle a patient’s sudden change in condition or explain things to the family. At the hospital where she was working at the time of the crime, the patient died suddenly. The family was absent at the time of death, and she encountered a scene where the nurse was severely blamed. She felt a strong sense of fear and did not want to be in a situation where she would be forced to respond during her working hours, so she committed crimes.

She had characteristics of ASD and was in a depressed state at the time of the offense. Although characteristics of ASD, such as the inability to handle multiple things at the same time, difficulty in dealing with interpersonal relationships, a narrow perspective on problem solving, and self-centeredness, were recognized, the criteria for the DSM-5 diagnosis were not met, and a definitive diagnosis of ASD could not be made.

The psychiatric evaluation was conducted based on case records, various test results, and the defendant’s school reports from elementary and junior high school, in addition to an interview with the defendant and two interviews with her parents, which allowed the evaluator to sufficiently understand episodes from early childhood. Considering the DSM-5 diagnostic criteria, the evaluator concluded that the defendant did not meet the criteria for ASD. The evaluator also found that although the defendant was considered to have been in a depressive state, she did not satisfy the diagnostic criteria for depressive disorder, given that she had been able to perform her duties as a nurse and was able to think through the steps required to carry out the offenses.

The judgment further stated that the defendant exhibited characteristics of ASD, and that she became depressed, accumulated stress, and fell into a narrowed perspective, leading her to a simplistic idea that eliminating the patients she was responsible for was the only way to reduce her anxiety, resulting in repeated offenses. The court held that this process of motive formation was strongly influenced by circumstances beyond the defendant’s ability to control and therefore should be regarded as circumstances in her favor.

On the other hand, the defendant selected the means of committing the offenses in accordance with her purpose of causing the victims to die at times when she did not have to attend to them, and she acted while taking care not to reveal her wrongdoing. She also recognized that her conduct was illegal and acted purposively. Therefore, even if the defendant’s autistic characteristics and depressive state are regarded as a mental disorder, the court determined that her ability to understand the wrongfulness of her conduct and her ability to control her behavior had not been significantly impaired.

In addition to the above psychiatric considerations, the judgment identified several non-psychiatric factors that strongly influenced the sentencing decision. The court found that the defendant acted in accordance with her purpose of causing the victims to die at times when she would not be required to respond as a nurse, and that she made use of her professional knowledge and position in selecting methods that would avoid detection. The judgment stated that these aspects demonstrated planning and deliberation. It further held that the risk to life was extremely high, that the defendant’s disregard for human life was severe and malicious, and that the offenses were particularly egregious.

The court also noted that the defendant committed the offenses so that the hospitalized patients would die before the next night shift, thereby allowing her to avoid interacting with their families. It described this motive as entirely self-centered and concluded that there were no further circumstances that could be counted in her favor.

Several circumstances were considered when determining the sentence. The circumstances surrounding the formation of her motive were strongly influenced by circumstances beyond her control, and this was taken into consideration in her favor. In addition, she honestly recounted her memories, including those that were unfavorable to her, and was deeply aware of the seriousness of the crime she had committed, expressing her remorse and saying, “I want to atone by dying.” Therefore, it was judged that she had the potential to rehabilitate herself and was sentenced to indefinite imprisonment instead of the death penalty sought by the prosecutor. The judgment document stated, “It is appropriate to make her face the seriousness of the crime she committed for the rest of her life, and to make her pay for her crime while also allowing her to walk the path of rehabilitation.”

#### 3.1.3 Other ASD-related criminal judgments in Japan

To contextualize the present cases, we also reviewed other ASD-related criminal judgments in Japan. While most defendants with ASD are judged to have full criminal responsibility, there are rare cases in which insanity or diminished responsibility has been recognized. For example, in cases where ASD co-occurred with brief psychotic disorder (8) or depressive disorder (9), the court accepted a finding of insanity. In another case, intrusive recollections of bullying and obsessive traits contributed to aggressive impulses, leading the court to recognize diminished responsibility (10). Additionally, an ASD-related homicide case in which the death penalty has been sought remains pending.

## 4 Discussion

The two Japanese court cases presented in this study illustrate how judicial reasoning regarding defendants with ASD or autistic traits can diverge dramatically, even though both crimes involved multiple victims and professional responsibilities in healthcare settings. In Case 1, the presence of a formal ASD diagnosis and low intellectual functioning were acknowledged, yet the defendant’s shifting statements and lack of remorse were interpreted as evidence of irremediable dangerousness. In contrast, Case 2 involved only autistic traits without a definitive diagnosis. Here, the court emphasized her consistent testimony, acknowledgment of wrongdoing, and expression of remorse, concluding that rehabilitation remained possible.

In both cases, the courts ultimately concluded that the defendants retained full criminal responsibility. In Case 1, despite a formal diagnosis of autism spectrum disorder and acknowledged psychological distress, the court determined that the defendant preserved both cognitive capacity (the ability to understand the wrongfulness of his actions) and volitional capacity (the ability to act in accordance with that understanding). This conclusion was based on the repeated and patterned nature of the offenses, evidence of planning and concealment, purposeful behavior, and maintained occupational functioning as a healthcare professional. These factors were considered decisive in supporting a finding of full responsibility.

Similarly, in Case 2, although autistic traits and a depressive state were acknowledged, the court found that the defendant retained sufficient cognitive and volitional capacities at the time of the offenses. The judgment emphasized her deliberate selection of means, the timing of the acts to avoid detection and professional obligations, and her clear awareness of the illegality of her conduct. On this basis, the court concluded that criminal responsibility was not significantly impaired. The sentencing outcome differed due to mitigating considerations related to motive formation, expressed remorse, and perceived rehabilitation potential, resulting in a sentence of indefinite imprisonment rather than the death penalty.

From a psychiatric perspective, these judgments highlight a paradox. Clinical features of ASD—such as difficulties in communication, restricted interests, or atypical expressions of emotion—can be misinterpreted in legal contexts as indicators of callousness, lack of humanity, or absence of remorse. Consequently, traits that stem from neurodevelopmental differences risk being conflated with moral failings, leading to harsher sentencing.

Recent empirical studies suggest that evaluations of remorse systematically disadvantage autistic individuals. For instance, a mock-juror study found that autistic defendants were often judged as less remorseful than non-autistic defendants, which in turn led to harsher punitive attitudes and sentencing recommendations (11). This supports our concern that reliance on remorse as a key criterion may disproportionately penalize individuals whose affective expression and communicative style differ from neurotypical norms.

Moreover, both judgments reveal a limited and unscientific framework for distinguishing between the death penalty and indefinite imprisonment. The central determinant was not psychiatric evidence of risk or rehabilitation potential, but rather the court’s subjective evaluation of “apology” and “remorse.” This reliance on affective display and narrative coherence as proxies for moral character may systematically disadvantage defendants with ASD, whose communicative styles differ from neurotypical expectations.

Research on courtroom perception has shown that atypical communicative behaviors—such as reduced eye contact, flat affect, or atypical prosody—can lead to reduced perceived credibility and heightened perceptions of culpability among jurors evaluating autistic defendants (12, 13). Such findings underscore the risk that autistic communication styles may be misinterpreted as deception, indifference, or lack of sincerity, rather than as manifestations of a neurodevelopmental condition.

Finally, systematic survey data reveal that autistic individuals remain under-recognized within criminal justice settings, and that standard procedures and accommodations are rarely implemented for them (14). This institutional neglect may exacerbate interpretive biases and further compromise fair treatment in trials and sentencing.

Considering these findings, the divergent judicial assessments in the two Japanese cases presented herein are not unique anomalies but reflect broader, internationally documented patterns of misunderstanding and structural bias toward autistic individuals in legal contexts.

In Japan, there is no standardized or validated framework for evaluating remorse in criminal proceedings. Judicial determinations rely largely on subjective impressions of a defendant’s demeanor and manner of expression. Although psychological instruments relevant to assessing attribution of responsibility have recently been developed, such as the Japanese version of the Gudjonsson Blame Attribution Inventory-Revised (15), these tools have not been validated for individuals with ASD and therefore cannot differentiate between ASD-related communication patterns and an actual absence of remorse.

As discussed earlier, ASD-related communication characteristics may cause autistic defendants to appear indifferent or unreflective even when these impressions arise from neurodevelopmental traits rather than their internal moral stance. These limitations highlight the need to reconsider how remorse is evaluated in legal contexts and to explore whether structured attribution or responsibility assessment tools could be adapted or validated for defendants with ASD.

These cases underscore the responsibility of forensic psychiatry to bridge the gap between psychiatric science and judicial practice. By clarifying how ASD affects behavior, cognition, and emotional expression, psychiatrists can contribute to fairer sentencing decisions and reduce the risk that death penalty determinations are made based on misinterpretation rather than evidence. Ultimately, these cases illustrate not only the life-and-death consequences of inadequate understanding of ASD, but also the vital role of forensic psychiatry in promoting justice, scientific accuracy, and respect for human dignity.

The present analysis has several implications for forensic practice and criminal justice policy. Experimental research shows that psychoeducation about ASD can improve perceptions of credibility, culpability, and apparent remorse among legal decision-makers (12, 13). In addition, system-level findings from international case analyses indicate that autistic individuals are often disadvantaged within criminal justice processes due to limited accommodations, inconsistent interpretation of autistic traits, and insufficient understanding among professionals (14). These results highlight the need for structured judicial training on neurodevelopmental disorders.

Moreover, Japanese courts do not currently employ standardized or validated methods for evaluating remorse. Although psychological instruments relevant to responsibility attribution exist (15), these tools have not been validated for individuals with ASD and therefore cannot distinguish ASD-related communication patterns from genuine absence of remorse. Developing guidelines for evaluating remorse in defendants with neurodevelopmental conditions is therefore an important next step.

Forensic psychiatrists also play a crucial role in communicating ASD-related nuances to the court. Clear explanations of how ASD affects emotional expression, narrative coherence, and courtroom demeanor—and of the limitations of existing assessment methods—may help reduce misinterpretation. Prior research demonstrates that improved understanding of ASD can attenuate biased judgments in legal contexts (12–14). Implementing these measures may contribute to more equitable and evidence-based sentencing practices.

## 5 Conclusion

This case note highlights the reliance on subjective assessments of remorse and the limited standardization in evaluating rehabilitation potential when distinguishing between the death penalty and indefinite imprisonment in defendants with ASD traits. Psychiatric expertise should contribute to fairer and more evidence-based sentencing.

## Data Availability

The original contributions presented in the study are included in the article/supplementary material. Further inquiries can be directed to the corresponding author.
